# The Readthrough Isoform AQP4ex Is Constitutively Phosphorylated in the Perivascular Astrocyte Endfeet of Human Brain

**DOI:** 10.3390/biom12050633

**Published:** 2022-04-25

**Authors:** Roberta Pati, Claudia Palazzo, Onofrio Valente, Pasqua Abbrescia, Raffaella Messina, Nicoletta Concetta Surdo, Konstantinos Lefkimmiatis, Francesco Signorelli, Grazia Paola Nicchia, Antonio Frigeri

**Affiliations:** 1 Department of Basic Medical Sciences, Neurosciences and Sense Organs, School of Medicine, University of Bari Aldo Moro, 70124 Bari, Italy; roberta.pati@uniba.it (R.P.); claudia.palazzo@uniba.it (C.P.); onofrio.valente@uniba.it (O.V.); pasqua.abbrescia@uniba.it (P.A.); raffaella.messina@uniba.it (R.M.); francesco.signorelli@uniba.it (F.S.); 2 Foundation for Advanced Biomedical Research, Veneto Institute of Molecular Medicine, 35129 Padova, Italy; nicolettaconcetta.surdo@cnr.it (N.C.S.); konstantinos.lefkimmiatis@unipv.it (K.L.); 3 Neuroscience Institute, National Research Council of Italy (CNR), 35129 Padova, Italy; 4 Department of Molecular Medicine, University of Pavia, 27100 Pavia, Italy; 5 Department of Bioscience, Biotechnology and Biopharmaceutics, University of Bari Aldo Moro, 70124 Bari, Italy; graziapaola.nicchia@uniba.it; 6 Dominick P. Purpura Department of Neuroscience, Albert Einstein College of Medicine, 840 Kennedy Center, Bronx, NY 10461, USA

**Keywords:** Aquaporin-4 (AQP4), *p*-AQP4ex, phosphorylation, translational readthrough, short-term regulation

## Abstract

AQP4ex is a recently discovered isoform of AQP4 generated by a translational readthrough mechanism. It is strongly expressed at the astrocyte perivascular endfeet as a component of the supramolecular membrane complex, commonly called orthogonal array of particles (OAP), together with the canonical isoforms M1 and M23 of AQP4. Previous site-directed mutagenesis experiments suggested the potential role of serine^331^ and serine^335^, located in the extended peptide of AQP4ex, in water channel activity by phosphorylation. In the present study we evaluated the effective phosphorylation of human AQP4ex. A small scale bioinformatic analysis indicated that only Ser^335^ is conserved in human, mouse and rat AQP4ex. The phosphorylation site of Ser^335^ was assessed through generation of phospho-specific antibodies in rabbits. Antibody specificity was first evaluated in binding phosphorylated peptide versus its unphosphorylated analog by ELISA, which was further confirmed by site-directed mutagenesis experiments. Western blot and immunofluorescence experiments revealed strong expression of phosphorylated AQP4ex (*p*-AQP4ex) in human brain and localization at the perivascular astrocyte endfeet in supramolecular assemblies identified by BN/PAGE experiments. All together, these data reveal, for the first time, the existence of a phosphorylated form of AQP4, at Ser^335^ in the extended sequence exclusive of AQP4ex. Therefore, we anticipate an important physiological role of *p*-AQP4ex in human brain water homeostasis.

## 1. Introduction

Aquaporin-4 (AQP4), the main water channel in the central nervous system (CNS), is abundantly expressed at the perivascular astrocyte endfeet close to the blood-brain barrier interface [1]. AQP4 is involved in the maintenance of cerebral water and ion balance and in blood-brain barrier development and integrity [2,3]. Recent studies also suggest a role for AQP4 in waste clearance by the glymphatic pathway [4]. AQP4 isoform expression is influenced by the 5′UTR and 3′UTR sequences of mRNA translation. Indeed, AQP4 is expressed as two main isoforms: a full-length, long isoform with translation initiation at Met-1 of 32 kDa (M1), and a shorter isoform produced by leaky scanning with translation initiation at Met-23 of 30 kDa (M23) [5]. These two isoforms associate first in heterotetramers, which further aggregate into well-ordered supramolecular structures [6,7,8] called orthogonal arrays of particles (OAPs) identified by freeze fracture electron microscopy [9,10]. A novel AQP4 isoform (AQP4ex) produced by a translational readthrough mechanism has been recently identified [11,12,13]. AQP4ex contains a 29-amino acid extension at the C-terminal essential to interact with α-syntrophin [12], a member of the dystrophin complex. AQP4ex-KO mice demonstrate that AQP4ex is crucial for the anchoring of AQP4 at the perivascular pole of astrocytic membranes, and it is also necessary to generate the neuromyelitis optica IgG epitope [14]. Importantly, mutagenesis experiments have demonstrated the phosphorylation of the two consensus serines (Ser^331^ and Ser^335^) located in the AQP4 readthrough extension [12].

Phosphorylation is a common post-translational modification that can contribute to the regulation of protein function by inducing structural changes and regulating protein-protein interactions [15,16]. The phosphorylation process is governed by a dense network of kinases and phosphatases, and is often tissue-specific [5], allowing proteins to be regulated based on the necessities of a specific tissue. In particular, we have reported that AQP4ex phosphorylation may have a role in water channel activity [12], suggesting a possible novel process in AQP4 short term regulation. However, AQP4 phosphorylation is still a controversial topic. Indeed, studies regarding phosphorylation-dependent gating and trafficking of AQP4 have generated contrasting results, most likely because of the use of the canonical isoforms (M1 and M23) in transfected cells [17]. In the present study, we analyzed the extended sequence of different species and selected the highly conserved Ser^335^ to produce a phospho-specific antibody against human AQP4ex. We then employed this antibody to assess its specificity and the existence, localization, supramolecular aggregation and level of phosphorylation of AQP4ex in the human brain. Our results show the expression of highly phosphorylated AQP4 at Ser^335^ of the extended portion of AQP4ex.

## 2. Materials and Methods

### 2.1. Multiple Alignment of Predicted Aquaporin Transcripts from Different Vertebrates and Mammals

The Entrez Gene database at the National Center for Biotechnology Information (NCBI) [18] was used for the predictions of orthologous and paralogue genes encoding AQP4ex in different species of vertebrates and mammals. The nucleotide sequences were translated to the corresponding peptide sequences using the Bioinformatic tool EMBOSS Transeq on the EMBL-EBI (European Bioinformatics Institute, Cambridge, EN) web site [19]. In order to identify the presence of consensus phosphorylation motif RXXS, a multiple amino acid sequence alignment was performed using Clustal Omega software [20] open access version 1.2.4 available on the EMBL-EBI website.

### 2.2. Cell Cultures and Transfection

Mouse astrocyte primary cultures were prepared as previously described [21]. Briefly, dissected neocortical tissues from newborn pups were minced, passed through a cell strainer (100 mesh) and incubated with 0.25% trypsin, 0.01% DNase in DMEM for 30 min at 37 °C. The suspension was centrifuged twice at 2100× *g* for 10 min. The pellet was resuspended and plated on T25 flasks in DMEM-Glutamax (Gibco, Hampton, NH, USA), 1% penicillin/streptomycin, 10% fetal bovine serum (FBS) and maintained at 37 °C in a 5% CO2 incubator. After 7–10 days in culture, flasks were shaken to remove microglia and oligodendrocytes, and astrocytes were seeded on 12 mm round glass coverslips for immunofluorescence and in 12 multiwell plates for immunoblot analysis. The HEK293 cell line was cultured in Dulbecco’s high glucose medium (Gibco, Hampton, NH, USA) supplemented with 10% FBS (Gibco, Hampton, NH, USA) and penicillin/streptomycin (Gibco, Hampton, NH, USA). The day before transfection, cells at 60 to 70% confluency were plated using Optimem antibiotic-free medium (Gibco, Hampton, NH, USA). Transfection was carried out with 1 µg of pT-AQP4ex^S331A/S335A^ phosphonull, pT-AQP4ex^S331D/S335D^ phosphomimetic and pT-AQP4ex [12]. Cells were transfected with Lipofectamine2000 (Invitrogen, Waltham, MA, USA) according to the manufacturer’s protocol and analyzed after 24 h.

### 2.3. Antibodies

A custom affinity-purified peptide polyclonal antibody was generated by GenScript Biotech (Piscataway, NJ, USA) against the human peptide containing the phosphorylated Ser^335^, within the C-terminal extension at a concentration of 0.2 μg/mL for immunoblot and at 0.5 μg/mL for immunofluorescence. A custom rabbit polyclonal anti human AQP4ex [12] was used at a concentration of 0.3 μg/mL for both immunoblot and immunofluorescence, and a custom rabbit anti-AQP4 [22] was used at a concentration of 0.13 μg/mL for immunoblot and at 0.4 μg/mL for immunofluorescence. For immunofluorescence on cells, an AlexaFluor 488 anti-rabbit was used as secondary antibody (Life Technologies, Thermo Fisher Scientific, Carlsbad, CA, USA) at a concentration of 1 µg/mL. For immunofluorescence on tissue sections, an AlexaFluor 594 anti-rabbit was used as secondary antibody (Life Technologies, Thermo Fisher Scientific, Carlsbad, CA, USA) at a concentration of 1 µg/mL. For immunoblotting, anti-rabbit IgG-HRP was used, following the manufacturer’s instructions (Bio-Rad, CA, USA). To stain vessels, Lycopersicon Esculentum Tomato LEA 488 (Life Technologies, Carlsbad, CA, USA) was used at a concentration of 2.4 μg/mL. For co-immunofluorescence on transfected cells a high titer NMO-IgG positive serum containing AQP4-IgG [23] was used at 1:50 dilution, and Alexa Fluor 594 anti-human as secondary antibody at a concentration of 1 µg/mL.

### 2.4. Human Brain Biopsies

Tissues were obtained from neurosurgery. Specimens of human tissues were obtained from surgical intraoperatively resection of the following categories of patients with glioblastoma multiforme. (a) A 55-years-old patient with fronto-temporo-insular glioblastoma. (b) A 53-years-old-patient with frontal glioblastoma. (c) A 54-years-old patient with temporal glioblastoma. The brain region analyzed represents unaffected cortical brain areas supplied with glioblastoma multiforme, and defined as the zone of brain parenchyma surrounding tumor tissue, apparently normal under the microscope. Details on these biopsies have been recently reported [22]. The study was conducted in accordance with the Principles of Ethics for Medical Research Involving Human Subjects set out in the Helsinki Declaration and its subsequent amendments and approved by the local institutional review board (project n. 6898).

### 2.5. Immunofluorescence

#### 2.5.1. Human Brain Tissue

Immunofluorescence experiments were performed as previously described [24]. Briefly, brain sections (10 μm) were fixed in 4% formaldehyde in PBS, washed with PBS, blocked using 0.1% gelatin in PBS for 15 min at room temperature, incubated at room temperature for 45 min with primary antibodies, washed with PBS-gelatin, and incubated for 40 min with Alexa Fluor 594 anti-rabbit secondary antibody. Vessels were stained with Lycopersicon Esculentum Tomato LEA 488 (Life Technologies, Carlsbad, CA, USA).

#### 2.5.2. Mammalian Cells

HEK293 cells were plated on round coverslips, fixed in 4% formaldehyde solution, washed with PBS, and permeabilized with 0.3% Triton X-100 in PBS. After blocking with 0.1% gelatin in PBS, the cells were incubated with primary antibodies for 40 min at room temperature. After washing in PBS, the cells were incubated with Alexa Fluor 488 anti-rabbit secondary antibody for 30 min. Finally, sections were viewed with a Leica DM2500 LED fluorescence microscope using 20×/0.55 and 40×/0.80 PL FLUOTAR objectives. Confocal images were obtained using an automated inverted Leica TCS SP8 confocal microscope with a 40× HC PL Apo oil CS2 objective for human brain slices, and on a ZEISS LSM900 with Airyscan 2–Super Resolution system using a Zeiss Plan-Apochromat 63×//1.4 Oil immersion objective for cell immunofluorescence. For AQP4-IgG (NMO) co-immunofluorescence, cells were seeded on round coverslips, washed with PBS and after blocking with 0.1% gelatin in PBS, were incubated with AQP4-IgG positive serum for 40 min at room temperature. Cells were washed, fixed, permeabilized and blocked as mentioned above and incubated with anti *p*-AQP4ex antibody for 40 min at room temperature. After washing in PBS, cells were incubated with Alexa Fluor 488 anti-rabbit and Alexa Fluor 594 anti-human secondary antibodies for 30 min. Coverslips were mounted with Mowiol (Sigma-Aldrich, Waltham, MA, USA) added with DAPI (4′,6-diamidino-2-phenylindole, Life Technologies, Thermo Fisher Scientific, Waltham, MA, USA). Images were obtained under an SP8 confocal automated inverted Lightning microscope (Leica TCS) using a 40X HC PL Apo oil CS2 objective.

### 2.6. Sample Preparation for SDS-PAGE

Harvested tissues were frozen in liquid nitrogen and stored at −80 °C for SDS-PAGE experiments [8]. Proteins were extracted from stored brain biopsies in 7 volumes of BN buffer (1% Triton X-100, 12 mM NaCl, 500 mM 6-aminohexanoic acid, 20 mM Bis–Tris, pH 7.0, 2 mM EDTA, 10% glycerol) added with an EDTA-free cocktail of complete protease inhibitors (Roche, Milan, Italy). Transfected HEK293 cells were washed once in ice-cold PBS and dissolved in RIPA buffer (10 mM Tris-HCl, pH 7.4; 140 mM NaCl; 1% Triton X-100; 1% Na+ deoxycholate; 0.1% SDS; 1 mM Na3VO4; 1 mM NaF and 1 mM EDTA) added with a cocktail of protease inhibitors (Roche, Milan, Italy). Cells and tissues lysates were incubated on ice for 30 min and centrifuged at 17,000× *g* for 40 min at 4 °C. The supernatants were collected, and the protein concentration was determined using the BCA protein assay (Thermo Scientific, Waltham, MA, USA). For dephosphorylation, 40 µg of lysates were treated with 200 U of Lambda protein phosphatase for 30 min at 30 °C (New England biolabs, Ipswich, MA, USA) according to the manufacturer’s protocol.

### 2.7. Sample Preparation for BN-PAGE

Human tissues were homogenized in 7 volumes of BN buffer (1% Triton X-100, 12 mM NaCl, 500 mM 6-aminohexanoic acid, 20 mM Bis–Tris, pH 7.0, 2 mM EDTA, 10% glycerol) added with EDTA-free complete protease inhibitor cocktail, (Roche, Basel, Switzerland). After lysis on ice for 1 h, samples were centrifuged at 17,000× *g* for 30 min at 4 °C. A BCA Protein Assay Kit (Thermo Scientific, Waltham, MA, USA) was used to determine the total protein content of supernatants. The BN-PAGE experiments were performed as previously described [8] using 60 μg of protein sample previously mixed with 2 μL of loading buffer (5% Coomassie Blue G-250, 750 mM aminocaproic acid) and 10% glycerol by volume. At the end of gel running, proteins were transferred to PVDF membranes (Millipore, Burlington, MA, USA) for immunoblot analysis, as described below.

### 2.8. SDS-PAGE and Western Blot Analysis 

Electrophoresis and immunoblotting were performed as previously described [8]. Briefly, proteins were separated on 13% SDS/PAGE and transferred to polyvinylidene difluoride (PVDF) membranes (Millipore, Burlington, MA, USA) for immunoblot analysis. Membranes were incubated with primary antibodies overnight, washed, and incubated with peroxidase-conjugated secondary antibodies at room temperature for 45 min. Reactive proteins were revealed using an enhanced chemiluminescent detection system (Clarity Western ECL Substrate, Bio-Rad, CA, USA) and visualized on a Chemidoc Touch imaging system (Bio-Rad, CA, USA).

## 3. Results

### 3.1. Multiple Alignment Analysis of AQP4 Extension

We have previously provided evidence that serine phosphorylation in the extended C-portion may play a crucial role in AQP4ex water transport function [12].

**Figure 1:**
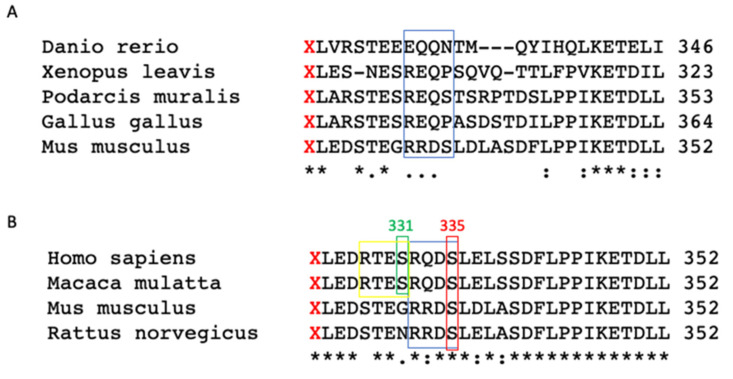
Multiple alignment of AQP4ex C-terminal extension in different vertebrates (**A**) and (**B**) in mammals. The asterisk (*) indicates a position with a single, fully conserved residue; colon (:) indicates conservation between groups of strongly similar properties; period (.) indicates conservation between groups of weakly similar properties. X in red highlights the canonical stop codon indicating the start site of translational readthrough extension. The box in red shows Ser^335^ preserved in mammals and inserted inside a consensus phosphorylation motif RXXS (blue box). The box in yellow indicates the consensus sequence RXXS, which includes Ser^331^ (green box) in Homo sapiens and Macaca mulatta.

To evaluate whether phosphorylation is actually occurring in AQP4ex and to determine which amino acid is involved in the C-terminal extension of human AQP4ex, we first conducted a DNA and protein search of different genomes. The analysis showed that the C-terminus extended sequence is conserved among the vertebrate subgroups: fish, reptiles, birds, amphibians and mammals (Figure 1A).

As previously reported, two serines with a high-score consensus phosphorylation (RXXS) motif [11] are present in the additional extended peptide. These two serines (Ser^331^ and Ser^335^) are only partially conserved among subgroups. In particular, among those analyzed, the wall lizard (Podarcis muralis) and the mouse have Ser^335^ with the consensus motif conserved. Instead, among the analyzed species, Ser^331^ was not present or included in the consensus motif. Thus, to investigate the role of phosphorylation within the extended sequence, a multiple alignment was limited to that between species among some mammals and primates. Interestingly, we found that only one phosphorylation motif is conserved, which relates to Ser^335^ (Figure 1B, red box) present in some mammals (mouse and rat) and primates (human and macaque) (Figure 1B, blue box). Furthermore, Ser^331^ is present in human and macaque with the consensus motif RXXS, whereas its phosphorylation site is not present in mouse or rat. Within the consensus motif, there are fully conserved residues among the different species of mammals, indicated with an asterisk (Figure 1B, blue box). However, in rodents, the amino acid substitution took place between residues having similar properties, indicated with a colon, by implementing so-called “neutral substitution” which allows the functionality of the protein itself to be kept unchanged.

### 3.2. Characterization of Antibodies on p-Ser^335^ Using Transfected Cells

Based on the highly conserved Ser^335^ in mouse, rat and human, we decided to generate antibodies using a phosphopeptide (DRTESRQD-PSer-LELSSC) specific for the human form of *p*-AQP4ex. Antibodies were purified by affinity chromatography using the phosphorylated peptide, and specificity evaluated by indirect ELISA against the phosphopeptide and unphosphorylated peptides (Table 1).

For the phosphopeptide, the limiting dilution was 1:512,000, whereas it was 1:8000 for the unphosphorylated peptide. These data demonstrate that anti *p*-AQP4ex is at least sixty-four-fold more specific for the phosphorylated peptide than the unphosphorylated one. Binding on native protein and specificity were evaluated in transfected cells using immunofluorescence. Specificity of the antibody was analyzed using HEK293 transiently transfected cells with the human WT-AQP4ex and with mutants in the two serines, Ser^331^ and Ser^335^, previously used to characterize the water channel activity of AQP4ex. In detail, the two serines were replaced with alanines to prevent phosphorylation (AQP4ex-^S331A/S335A^, phosphonull) and with aspartates (AQP4ex^S331D/S335D^, phosphomimetic) in an attempt to change the phosphorylation of serines in the AQP4ex C-terminal extension.

**Figure 2:**
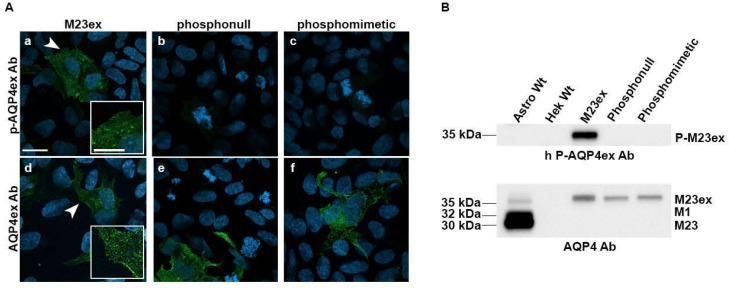
Characterization of *p*-AQP4ex antibody. Specificity of the antibody to the AQP4ex extension was analyzed by immunofluorescence on transfected cells (**A**) with constructs expressing M23ex (**a**,**d**), AQP4ex-phosphonull (**b**,**e**), or AQP4ex-phosphomimetic (**c**,**f**). Cells were stained with anti-*p*-AQP4ex and anti-AQP4ex antibodies. Note that the phosphorylated AQP4ex signal is observable only in AQP4ex expressing cells but not in cells expressing each mutant. Scale bar 20 μm. Insets show at higher magnification the region indicated by white arrowhead. Scale bar 10 μm. (**B**) Top, immunoblot analysis with specific phospho-antibody revealed the phosphorylated form of AQP4-M23ex (*p*-M23ex) at the predicted size (35 kDa) only in cells expressing the wild type human AQP4-M23ex (M23ex) and not in other cell lysates. Bottom, anti-AQP4 antibody recognized a band at 35 kDa in transfected cells with each construct. In astrocyte lysates, three bands of 35, 32, and 30 kDa were detected that corresponded to AQP4-M23ex, AQP4-M1, and AQP4-M23, respectively.

The antibody was able to recognize *p*-AQP4ex in cells expressing WT-AQP4ex, while both mutations affected *p*-AQP4ex antibody binding (Figure 2A). Proper expression and binding to the mutated proteins were verified using the AQP4ex antibody (Figure 2A). These data show that the antibody only specifically recognizes the human AQP4ex when it is phosphorylated on Ser^335^ without any cross reactivity with the phospho-mutants, and on non-transfected cells. Occasionally, some non-specific staining was observed in dividing cells. This could be explained by the high number of phosphorylation events taking place in this process [25]. Specificity of the staining was also demonstrated using transfected cells co-stained with AQP4ex and NMO antibodies (see Supplementary Figure S1). Specificity of the antibody was also analyzed by immunoblot using transfected cells. The phospho-antibody was able to recognize the WT-AQP4ex of the predicted size (35 kDa) in control transfected cell lysates (Figure 2B and Supplementary Figure S2) while the signal was absent in the protein extract from cells transfected with each mutant, indicating high specificity of the antibody to the phosphorylated human extended isoform at Ser^335^. The species-selective binding of the antibody against the human phosphorylated isoform was confirmed by the absence of the signal in mouse astrocyte lysates (Figure 2B and Supplementary Figure S2).

### 3.3. p-AQP4ex Expression in Human Brain

**Figure 3:**
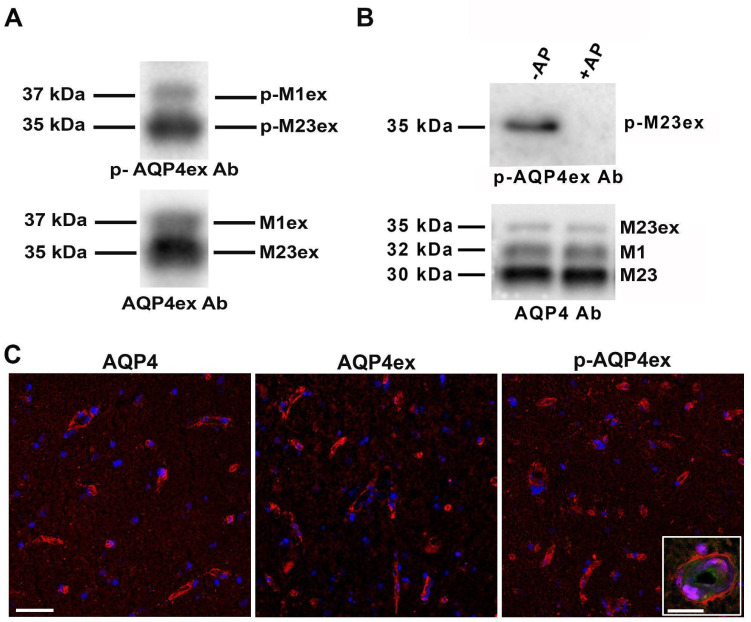
Expression and localization of *p*-AQP4ex in human brain cerebrum. (**A**) Immunodetection of the extended isoforms, respectively, M23ex (35 kDa) and M1ex (37 kDa), revealed with *p*-AQP4ex (top) and AQP4ex antibodies (bottom) shows that both extended isoforms are phosphorylated. (**B**) Immunoblot analysis showing (top) the expression of *p*-AQP4ex in untreated brain lysate (−AP), and the complete abolishment of the signal after alkaline phosphatase (AP) treatment (+AP). Bottom, levels of AQP4 isoform expression showing that the expression of AQP4 is not affected after treatment. (**C**) Localization of p-AQP4 isoform in human brain tissue. Note that *p*-AQP4ex is localized at the perivascular pole as AQP4 and AQP4ex. Perivascular staining of *p*-AQP4ex is observable at higher magnification and confocal microscopy (inset) and was confirmed by lectin staining (green) used as a blood vessel marker. Scale bar 50µm. Inset scale bar 20 µm.

Human AQP4ex is strongly expressed in the perivascular astrocyte processes of the human brain [22]. Immunoblot and immunofluorescence experiments using human brain unfixed cryosections were conducted to evaluate binding capacity, localization and level of expression of *p*-AQP4ex. From immunoblot analysis, we found that both the extended isoforms are expressed in the human cerebrum and are highly phosphorylated at Ser^335^ (Figure 3A). The specificity of these results was demonstrated by treating the homogenate with alkaline phosphatase (Figure 3B).

We have previously reported that AQP4 and AQP4ex show perivascular staining both in mouse [14] and human brain [22]. In order to evaluate possible differences in the localization of *p*-AQP4ex, we conducted immunofluorescence experiments on human brain cryosections. *p*-AQP4ex staining showed the typical pattern of AQP4ex expression (Figure 3C), and localization at the perivascular astrocyte endfeet (Figure 3C, inset), as confirmed by the blood vessel marker lectin.

### 3.4. p-AQP4ex Supramolecular Organization in Human Tissues

**Figure 4:**
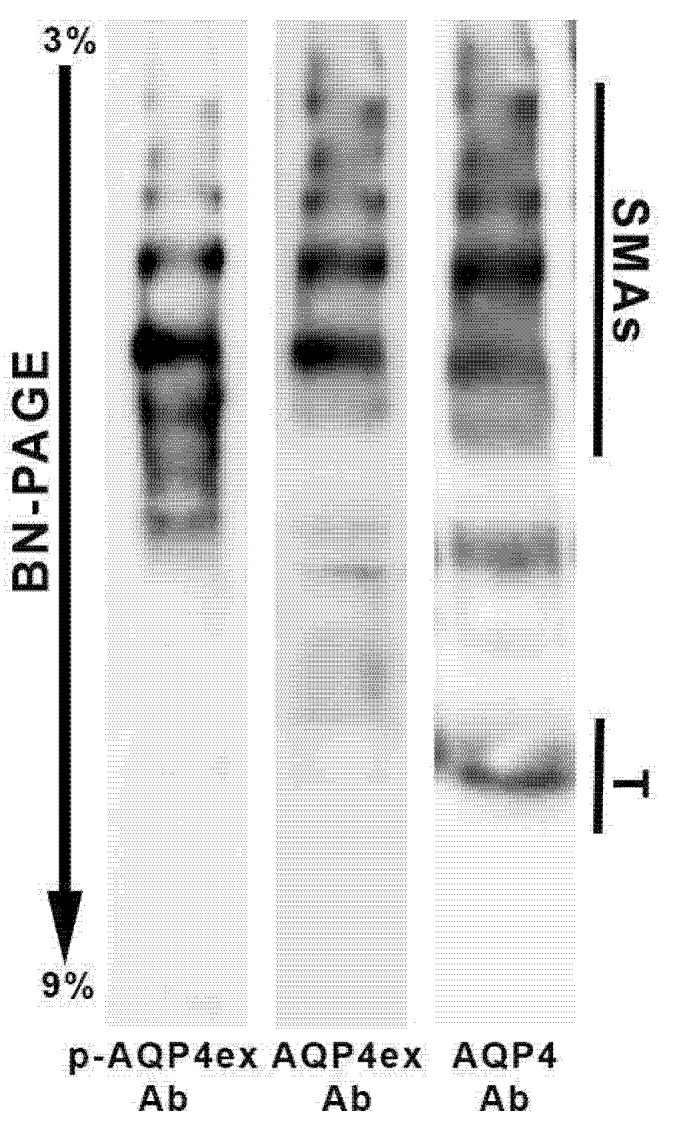
*p*-AQP4ex supramolecular organization in human brain. BN-PAGE experiments on human brain biopsy homogenates show that *p*-AQP4ex is expressed mainly in large size supramolecular assemblies (SMAs). A similar profile was revealed by the AQP4ex antibody. Note that the global AQP4 antibody reveals both high molecular weight pools (SMAs) and tetrameric forms of AQP4 (T).

BN-PAGE experiments were performed to determine whether *p*-AQP4ex is present in supramolecular assemblies (SMAs) (Figure 4). The phosphoantibody revealed the expression of *p*-AQP4ex in large sized AQP4 pools in the human brain, similar to that found in mice [14]. The number of pools expressing *p*-AQP4ex and AQP4ex appeared to be similar. However, some differences between AQP4ex and *p*-AQP4ex were found in terms of the intensity of the revealed pools, indicative of different amounts. These data suggest that AQP4ex is constitutively phosphorylated in SMA.

## 4. Discussion

In the present study, we describe for the first time the identification of a functionally active phosphorylation site of AQP4 located in the extended portion of the human AQP4ex. Several phosphorylation-dependent pathways have been proposed for AQP4 water flux regulation, trafficking and subcellular localization [14,26]. In line with these, various AQP4 sites have been proposed to be phosphorylated, including Ser^111^ in the cytoplasmic loop B [27] and Ser^180^ in loop D [28], which have been proposed to regulate channel gating. However, this is still a subject of controversy as also demonstrated by the example of Ser^111^, which has been reported by other authors as not involved in AQP4 phosphorylation and water transport regulation [29]. This controversy is probably due to the fact that most of the studies conducted to identify the putative phosphorylation site of AQP4 were carried out in transfected cells using canonical isoforms (M1 and M23) [13,15,17,29,30] or fragments derived from the canonical isoforms [31,32].

We recently reported the potential role in water channel activity of two serines (Ser^331^ and Ser^335^) located in the extended portion of human AQP4ex [12]. Nevertheless, in a preliminary bioinformatic analysis, we observed that only Ser^335^ appeared to be conserved in primates and rodents (see Figure 1). Analyzing the consensus sequence of Ser^335^ in primates, it is evident that the preserved RQDS sequence is partially modified in rodents containing a substitution of glutamine (Q) with arginine (R). However, this modification belongs to a group of amino acids with similar properties, suggesting that in the course of evolution it was necessary to preserve the specific R-Q/R-DS sequence for an important physiological function. This phosphorylatable site could be modulated by a specific kinase whose selectivity is determined by residues close to the serine [33]. In light of this, we supposed that Ser^335^ is most likely to be the principal candidate for human AQP4ex phosphorylation. Thus, to verify this hypothesis we generated an antibody for the phosphorylated Ser^335^. Using several approaches we show here, for the first time, that AQP4 is constitutively phosphorylated in the human brain through the expression of AQP4ex at Ser^335^. Immunofluorescence experiments showed that *p*-AQP4ex is mainly expressed at the astrocyte processes surrounding brain vessels, overlapping the location of AQP4ex. The phosphorylation shown by immunoblot and immunofluorescence suggest an important role for post-translational modification in the basal condition. Mutational studies indicate that phosphorylation of human AQP4ex may decrease water channel activity [12]. Thus, we speculate that this post-translational modification could modulate AQP4 activity at the perivascular pole of the blood-brain barrier, which should have functional consequences both at the physiological and pathological levels. It is of importance that AQP4 polarity depends on AQP4ex, as demonstrated in AQP4ex-KO mice and in glioblastoma [14,22]. To this end it will be necessary to investigate the phosphorylation pathways that determine changes in phosphorylation of AQP4ex and their consequences on the function and structure of AQP4.

BN-PAGE experiments showed the presence of *p*-AQP4ex in SMAs (i.e., OAPs) indicating that this modification could have a role in AQP4ex localization/anchoring at the perivascular pole and directly affect AQP4 complex stability. In the light of a recent study in which we reported that AQP4ex is critical in triggering the progressive mislocalization and downregulation of AQP4 in glioblastoma [22], it will be of interest to evaluate whether *p*-AQP4ex is involved. Furthermore, it will be of interest to evaluate if *p*-AQP4ex is involved in gliosis, as recently reported for AQP4ex [34].

Although Ser^331^ is not conserved in mouse and rat, it cannot be excluded that this second serine may play a role in human AQP4 as a second level of regulation by phosphorylation of this important region of AQP4. Further studies are required to answer this important point.

## 5. Conclusions

In conclusion, we have shown that AQP4ex is highly phosphorylated at Ser^335^ in the human brain. Similar to non-phosphorylated AQP4ex, *p*-AQP4ex is organized in supramolecular assemblies at the perivascular astrocytic domain abutting brain microvessels. Our data suggest that *p*-AQP4ex is part of the molecular functional unit of AQP4 at the blood-brain barrier level.

## Figures and Tables

**Table 1:** ELISA results of pre-immune serum, phospho-specific antibody and antibody against both modified and unmodified peptides. The titer is the highest dilution with S/B (Signal/Blank) ≥ 2.1, and the OD450 in blank is the average of two technical replicates. The starting concentration of 1 mg/mL and the corresponding dilution ratio was calculated based on the actual concentration. NC is negative control (pre-immune serum).

**Concentration** **(ng/mL)**		NC	1000.00	500.00	250.00	125.00	62.50	31.25	15.62	7.81	3.90	1.95	Blank	/	/
	**Diluition**	1:1000	1:1000	1:2000	1:4000	1:8000	1:16,000	1:32,000	1:64,000	1:128,000	1:256,000	1:512,000	Blank	Titer	Coating
**Sample**															
**Specific Antibody**		0.155	2.810	2.789	2.748	2.733	2.672	2.604	2.442	2.077	1.630	1.167	0.064	>1:512,000	A
		0.118	0.528	0.336	0.200	0.128	0.094	0.076	0.075	0.074	0.067	0.06	0.059	1:8000	B
**“Pan” Antibody**		/	2.765	2.705	2.684	2.683	2.579	2.368	2.147	1.691	1.196	0.799	0.070	>1:512,000	A
		/	2.786	2.659	2.613	2.584	2.491	2.327	2.045	1.547	1.127	0.707	0.066	>1:512,000	B

## Data Availability

Not applicable.

## Supplementary Materials

The following supporting information can be downloaded at: https://www.mdpi.com/article/10.3390/biom12050633/s1, Figure S1: Characterization of *p*-AQP4ex antibody, co-immunofluorescence. Figure S2: Characterization of *p*-AQP4ex antibody, western blots.
