# Supplementary material for: The Readthrough Isoform AQP4ex Is Constitutively Phosphorylated in the Perivascular Astrocyte Endfeet of Human Brain
Source: Biomolecules. 2022 Apr 25;12(5):633. doi: 10.3390/biom12050633 (PMC9138620; doi:10.3390/biom12050633)
Supplement: Supplementary file 1 [file biomolecules-12-00633-s001.zip › biomolecules-1652415-supplementary.pdf]

biomolecules

MDPI

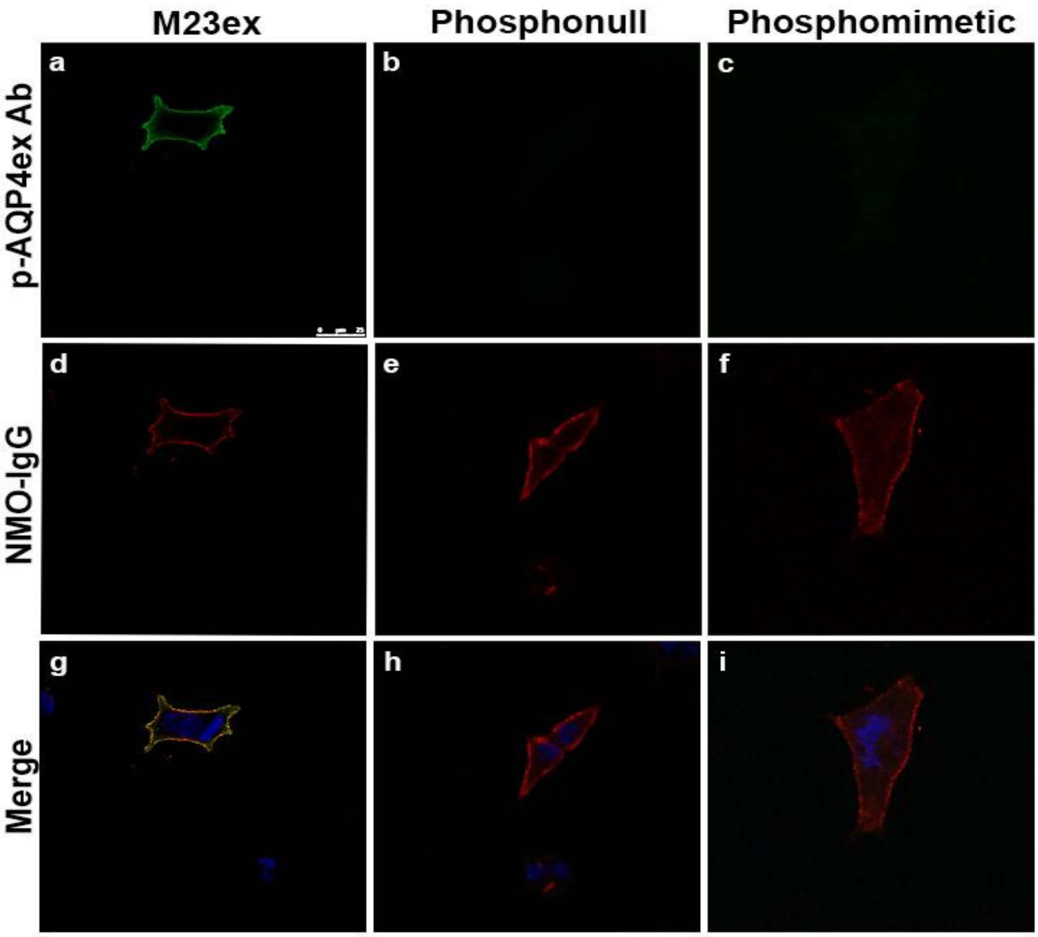

Figure S1. Characterization of p-AQP4ex antibody. HEK293 transfected cells were co-stained with p-AQP4ex and NMO-IgG antibodies. A Serum from NMO-IgG-positive patient was used to highlight the presence of AQP4 on the cell membrane. The phosphorylate AQP4ex signal is observable only in transfected cells with construct expressing M23ex (**a**) but not in cells expressing AQP4ex-phosphonull (**b**) and AQP4ex-phosphomimetic (**c**). Merge shows the colocalization of AQP4 and p-AQP4ex (**g**) in cells transfected with the isoform M23ex but not in the others (**h, i**). Nuclei are stained with DAPI. Scale bar 25μm.

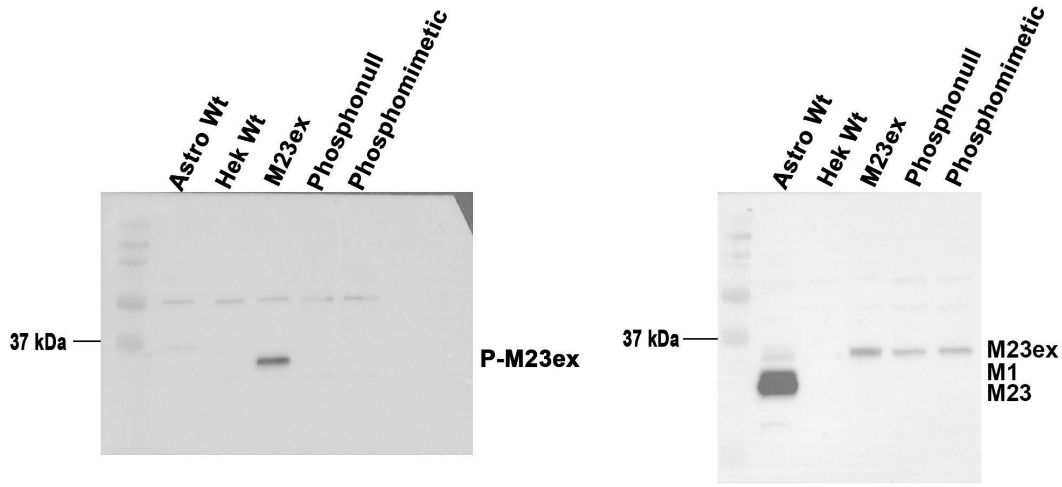

Figure S2. Characterization of p-AQP4ex antibody. Figure shows the whole blots of transfected cells lysates stained with anti-P-AQP4ex (left panel) and global AQP4 antibody (right panel).
